# Lornoxicam Side Effects May Lead to Surgical Mismanagement, in Case of Postoperative Intra-Abdominal Collection: A Case Report and Literature Review

**DOI:** 10.1155/2015/545807

**Published:** 2015-08-31

**Authors:** Mohammad Bukhetan Alharbi

**Affiliations:** Department of Surgery, Medical College, Al Imam Mohammad Ibn Saud Islamic University (IMSIU), P.O. Box 5701, Riyadh 11543, Saudi Arabia

## Abstract

Postoperative collection is a known complication of abdominal surgery, especially after major surgery; however, minor surgical procedures may also be associated with this phenomenon. Utilization of nonsteroidal anti-inflammatory drugs, such as lornoxicam, and the adverse effects thereof, may affect the surgeon's judgment regarding the need for, and extent of, draining of these collections. Here I report the case of a 25-year-old male who presented with perforated acute retrocaecal subhepatic appendicitis complicated by pleural effusion and a small abdominal collection. The pleural effusion resolved almost completely over time. However, the patient showed incomplete recovery, as demonstrated by nausea, vomiting, and mood disturbance along with abdominal pain, tachycardia, and a persistent small abdominal collection. We initially suspected infection caused by a highly virulent type of bacteria and planned to perform percutaneous drainage. However, owing to skin erythematic changes, administration of lornoxicam was ceased, which resulted in complete recovery of the symptoms and consequently in avoidance of unnecessary invasive intervention to drain the abdominal collection. These findings suggest that the utilization and adverse effects of some painkillers for postoperative pain, such as lornoxicam, may affect the surgeon's judgment regarding the most appropriate surgical workup in cases of postoperative fluid collection.

## 1. Introduction

Postoperative complications are relatively common in surgical practice, and, on most occasions, the surgeon can effectively manage these based on his or her prior experience. However, uncontrolled events may occur secondary to external factors, such as the use of certain drugs, which may affect the clinical judgment of, and provide more challenges to, the surgeon by requiring unnecessary interventions.

We here report on a 25-year-old male presenting with perforated acute retrocaecal subhepatic appendicitis, which was managed by open appendectomy and complicated by superficial surgical site infection, pleural effusion, and a moderate-size abdominal collection approximately one week after surgery, in whom the use of lornoxicam affected the surgeon's judgment regarding the need for draining of the postoperative collection.

## 2. Case Presentation

A 25-year-old male, who was otherwise healthy, presented to the emergency department of a private hospital, complaining of right lower abdominal pain along with nausea; the patient denied any similar attacks in the past, and there was no history of diarrhoea, changes in bowel habits, dyspepsia, heart burn, regurgitation, dysuria, or previous abdominal surgery.

Clinically, the patient appeared in mild pain, with a pulse rate of 90 beats/min and a temperature of 37.8 degrees Celsius. The systemic examination was unremarkable, whereas the abdominal examination revealed tenderness in the right lower quadrant. Laboratory investigations revealed a white blood cell count of 10,000/*μ*L, with neutrophils present. All electrolytes were within the normal limits, and the urine analysis findings were unremarkable.

The patient was diagnosed with perforated acute retrocaecal subhepatic appendicitis, and open appendectomy was consequently performed. Irrigation and suction were performed until clear effluent was obtained. The wound was left open to allow delayed primary closure. Postoperatively, the patient was managed by piperacillin/tazobactam and metronidazole. He initially showed significant improvement but was still unwell. On postoperative day 5, he developed dyspnoea, hypoxia, and fever. Computed tomography (CT) of the chest and abdomen showed a small fluid collection in the retrocolic space (7 × 6 cm in size) without enhancement and right pleural effusion. Despite resolution of the pleural effusion over the next 5 days, repeat CT showed persistence of the small residual abdominal collection in the same location.

The patient was readmitted to the hospital on postoperative day 9 with abdominal pain, nausea, tachycardia, and mood changes. The clinical workup revealed mild grade fever of 38.2, with pule rate of 90 beat/minute. Physical examination revealed superficial wound infection and his white blood cell count was 13,000/*μ*L, with no available PCR in the facility, but his CT abdomen showed a small abdominal collection. the patient was administered amoxicillin/clavulanic acid 675 mg PO Q8 h and lornoxicam 8 mg PO BID to overcome the infection process and pain. Accuracy of medications timing and dosage were confirmed. During admission, the symptoms persisted, whereas no significant clinical signs and a normal complete blood count were noted.

Despite its relatively minute amount, the abdominal collection was considered a concern due to the observed signs of infection, and percutaneous drainage was hence planned. However, there was an unexpected delay of CT-guided percutaneous drainage owing to reluctance of the patient. During this period, we moreover started to notice a large skin erythema on one of the patient's legs. This change was assessed by a dermatologist who consequently prescribed a lotion. However, no improvement was observed, and we instead changed the medical regimen by excluding lornoxicam, as skin rashes are a known side effect of this drug. This immediately (within 24 hours) resulted in the patient feeling well again, in cessation of his abdominal pain, vomiting, and lethargy, and in disappearance of the skin rash. Taken together, this suggests that medication use, such as lornoxicam in this case, can cause this unusual clinical scenario. Cessation of lornoxicam resulted in the patient recovering completely, not only in terms of his symptoms, but also in terms of the surgical site infection and the questionable abdominal collection.

## 3. Discussion

Medical care has developed dramatically over the last few decades, though with higher costs. Misdiagnosis was responsible for almost 20% of the mortality in one study using autopsies to confirm the cause of death [1].

Some drugs affect patient care in the clinic. Some cardiac, epilepsy, and hypoglycaemic medications, plus other drugs, have been shown to have an adverse impact on patient management [2–5].

The most common errors in medication use are either not giving the medication at the right time or not giving it at all [6]. Despite the many steps taken in health-care systems to ensure accurate medication use, there are still system failures [7].

Lornoxicam was introduced to the market in the 1990s. Data from preliminary clinical trials suggest that lornoxicam is as effective as the opioid analgesics morphine, pethidine (meperidine), and tramadol in relieving postoperative pain following gynaecological or orthopaedic surgery, and it is as effective as other NSAIDs after oral surgery. Lornoxicam is also as effective as other NSAIDs in relieving the symptoms of osteoarthritis, rheumatoid arthritis, ankylosing spondylitis, acute sciatica, and low back pain [8].

Current data support lornoxicam use for short-term pain control after a surgical procedure. The use of lornoxicam for other conditions, like headache, back pain, and sports injuries, has not been well studied. Complications related to gastrointestinal bleeding and cardiovascular disease have been reported [9].

COX inhibitors, like lornoxicam, work as antipyretic agent that might function as inhibitors for certain protease enzymes thought to mediate host cardiovascular response during cardiovascular responses during bacterial sepsis [10]. The first human study examining the role of COX inhibitors in sepsis was conducted by Haupt et al. [11]. This randomised, double blinded, multicentre study included 29 patients with clinical evidence of severe sepsis (16 were given ibuprofen and 13 were administered placebo). Eight of the ibuprofen treated patients presented with shock and seven had acute respiratory distress syndrome (ARDS), while four of placebo treated subjects had shock and four had ARDS. Nine patients in the COX inhibitor group died (56%) versus four in the placebo group (31%) [11]. Data suggest that in addition to any effects of host defence mechanisms against live pathogen, COX metabolites increase the mortality resulting from an overwhelming host inflammatory response, possibly due to their importance in systemic vasodilatation and renal blood flow [12].

The lack of clear benefits for COX inhibitors in human studies raise the question of the expected benefits of investigating prostaglandin synthesis pathway in the treatment of sepsis. Another explanation to the lack of benefits of COX inhibitors in human sepsis is that specific prostanoid molecules might need to be targeted, as opposed to blocking the most proximal committed step in prostaglandin synthesis [13].

From other sides, Memiş et al. reported intravenous lornoxicam to exert no effect on hemodynamic and biochemical parameters, cytokine levels, or patient outcomes in the immune-compromised patients with severe sepsis [14].

Other possible adverse effects of lornoxicam use have been reported, like hair loss [15] and lung inflammatory response syndrome [16]. Lornoxicam enhances the effect of glibenclamide due to its displacement of glibenclamide from its protein-binding site. It increases the concentration of warfarin leading to increased coagulation time. Lornoxicam decreases the clearance of digoxin from plasma and increases methotrexate concentrations [17].

There is nothing in the available literature discussing the use of lornoxicam for pain control in postoperative patients with infections. There is no explanation of the constitutional symptoms of lornoxicam and its possible conflict with the systemic response to infection, like nausea, vomiting, tachycardia, and lethargy.

## 4. Conclusion

The use of lornoxicam after abdominal surgery with complications may lead to constitutional symptoms that can mimic sepsis. Hence, the surgeon must have an ability to appreciate the correct clinical condition.
